# Swedish general practitioners’ attitudes towards treatment guidelines – a qualitative study

**DOI:** 10.1186/s12875-014-0199-0

**Published:** 2014-12-16

**Authors:** Veronica Milos, Tommy Westerlund, Patrik Midlöv, Eva Lena Strandberg

**Affiliations:** Department of Clinical Sciences in Malmö, Lund University, Malmö, Sweden; Medical Products Agency, Department of Medicine Usage, Uppsala and Sahlgrenska Academy, Department of Public Health and Community Medicine, Unit of Social Medicine, Institute of Medicine, University of Gothenburg, Gothenburg, Sweden; Department of Clinical Sciences in Malmö, Center for Primary Health Care Research, Lund University, Clinical Research Centre (CRC), Skåne University Hospital, building 28, floor 11, Jan Waldenströms gata 35, 205 02 Malmö, Sweden

**Keywords:** Qualitative research, Focus groups, Guidelines, Attitudes, Primary care, GPs, Adherence, Drug therapy

## Abstract

**Background:**

Drug therapy in primary care is a challenge for general practitioners (GPs) and the prescribing decision is influenced by several factors. GPs obtain drug information in different ways, from evidence-based sources, their own or others’ experiences, or interactions with opinion makers, patients or colleagues. The need for objective drug information sources instead of drug industry-provided information has led to the establishment of local drug and therapeutic committees. They annually produce and implement local treatment guidelines in order to promote rational drug use. This study describes Swedish GPs’ attitudes towards locally developed evidence-based treatment guidelines.

**Methods:**

Three focus group interviews were performed with a total of 17 GPs working at both public and private primary health care centres in Skåne in southern Sweden. Transcripts were analysed by conventional content analysis. Codes, categories and themes were derived from data during the analysis.

**Results:**

We found two main themes: *GP-related influencing factors* and *External influencing factors*. The first theme emerged when we put together four main categories: *Expectations and perceptions about existing local guidelines*, *Knowledge about evidence-based prescribing*, *Trust in development of guidelines*, and *Beliefs about adherence to guidelines.* The second theme included the categories *Patient-related aspects*, *Drug industry-related aspects*, *and Health economic aspects*. The time-saving aspect, trust in evidence-based market-neutral guidelines and patient safety were described as key motivating factors for adherence. Patient safety was reported to be more important than adherence to guidelines or maintaining a good patient-doctor relationship. Cost containment was perceived both as a motivating factor and a barrier for adherence to guidelines. GPs expressed concerns about difficulties with adherence to guidelines when managing patients with drugs from other prescribers. GPs experienced a lack of time to self-inform and difficulties managing direct-to-consumer drug industry information.

**Conclusions:**

Patient safety, trust in development of evidence-based recommendations, the patient-doctor encounter and cost containment were found to be key factors in GPs’ prescribing. Future studies should explore the need for transparency in forming and implementing guidelines, which might potentially increase adherence to evidence-based treatment guidelines in primary care.

## Background

Drug therapy in primary health care is a large field and a challenge for the medical world, pharmacists, related authorities and, most important of all, patients. The elderly population is increasing and so therefore is the need and importance of safe pharmacotherapy, with a focus on evidence-based medicine.

The broad skills of Swedish general practitioners (GPs) allow them to manage a vast spectrum of diseases and problems, with care accounting for patients’ complex needs. Following evidence-based medicine principles while maintaining the holistic view of the individual without risking patient safety are aspects a GP needs to consider in every prescribing decision. The challenge of continuously improving drug therapy while also meeting increasing pharmaceutical costs has resulted in both national and regional reforms in Sweden. These reforms include prescribing guidance and financial incentives in order to improve adherence to drug therapy recommendations [1]. Evidence-based treatment guidelines have been developed and are available for both primary and secondary care in Sweden.

GPs work in a broad medical field and therefore have a complex way of seeking medical information, with more direct patient-oriented care questions, which might differ from those of colleagues in other specialities who search for information from journals and other literature or by corresponding with colleagues [2]. However, GPs also base their decisions on “mindlines”, which are collectively reinforced, internalized, tacit guidelines, developed from own experiences or from interactions with colleagues, patients or pharmaceutical industry representatives [3]. This suggests that both formal and informal networking might influence prescribing behaviour.

Although GPs are aware of the guidelines, clinical inertia can lead to a conservative attitude [4]. Prescribing behaviour can vary a lot and the causes of the variation can be complex.

Unlike GPs in other European countries such as the Netherlands, Denmark and Norway, Swedish GPs work in public or tax-financed private multidisciplinary surgeries with several physicians, registered nurses and physiotherapists. Each surgery is given economic responsibility by the county council. While the structure of primary care demands financial responsibility on the part of physicians, there are efforts to meet patients’ needs and wishes and also to increase confidence in GPs. Due to the patient-centred approach used in Swedish primary care during recent decades, non-medical factors can influence the prescribing decision, such as organisation structure or patient age and gender [5]. Another aspect is that although GPs believe that costs should be taken into account when prescribing, they are considered secondary to clinical effectiveness and safety, whilst individual patient need is emphasized above other forms of rationality or notions of opportunity costs. Conflict might be apparent between a policy of cost containment and GPs’ resistance to cost-cutting [6]. At the same time, influences from both patients and the pharmaceutical industry put pressure on the doctor [7]. An interesting phenomenon is that physicians deny changing their prescribing habits according to patients’ wishes as a result of advertising from the pharmaceutical industry addressed directly to the public, but feel pressure to justify their prescribing habits [8]. Meanwhile, doctors with the most visits per week are the most likely to prescribe medicines according to patients’ wishes, even though they do not consider finding a medical reason for that [9]. Another important aspect is that GPs in Sweden do not have a gatekeeper role and the patients are free to consult other specialists without a referral [10]. The patients’ drug list might thus contain drugs prescribed by several physicians. According to the regulations of the Swedish National Board of Health and Welfare, the GPs have the responsibility for their own prescribed drugs, but should even, if possible, inform themselves about other drugs that the patient uses and assess whether the current prescription is appropriate [11]. However, GPs’ understanding of responsibility for patients’ medication lists varies [12] and lower adherence to medication guidelines could potentially arise. The prescribing decision is therefore multifaceted and strategies that influence prescribing patterns must take the abovementioned underlying factors into account.

In the Swedish county of Skåne, the local drug and therapeutic committee (DTC) develops treatment guidelines and publishes an annual list containing recommended drugs based on medical evidence but also economic considerations. The DTC works within multidisciplinary networks including GPs, secondary-care specialists, district nurses and pharmacists. The networks provide medication guideline lists for different specialities such as urology, psychiatry and dermatology, and sub-specialties of internal medicine such as endocrinology and ischemic heart diseases. They present the guidelines in a small booklet. More detailed background information is available in print and on the internet. Each network includes at least one GP. There is a special section for drug therapy in the elderly, including dosage reduction recommendations and a list of potentially inappropriate medications in elderly patients. This is especially important since multi-morbidity and polypharmacy are common in the elderly, which means that multiple treatment guidelines have to be taken into account.

In addition to the published list, the guidelines are also spread through academic detailing at primary care centres and an annual local informative conference.

There is no clear evidence that locally developed guidelines have a better effect on GPs’ adherence to evidence-based medicine compared to national guidelines, and there is an ongoing debate in Sweden as to whether the DTCs should focus on a consensus national list instead of each providing one list [13]. However, the role of knowledge exchange through professional networking has been suggested to be an important factor for transferring evidence into practice [14].

To increase compliance with local treatment guidelines, it is important to get a deeper understanding of GPs’ attitudes to them. Previous Swedish research has explored Swedish GPs’ attitudes towards evidence-based guidelines in general using focus groups as the study approach [15]. The aim of this study was to explore GPs’ attitudes towards locally developed treatment guidelines and the factors that affect adherence.

## Methods

In previous studies we assessed the effects of different intervention methods on GPs’ adherence to medication guidelines [16,17]. The qualitative design of the present study was chosen in order to get a deeper understanding of Swedish GPs’ attitudes towards local guidelines.

Focus groups have been widely used as an effective technique to explore the attitudes and needs of medical staff [18]. The method uses open-ended questions, allowing participants to approach the studied issues from a personal point of view. However, the debate within the group facilitates expression of beliefs and attitudes left undeveloped in an individual interview.

For practical reasons we chose to invite pre-existing focus groups of GPs with different experiences and genders, working at both private and public health care centres. The GPs did not interact with each other on a daily basis but had regular meetings every month.

Formal approval was obtained from the local DTC, which develops and publishes treatment guidelines annually.

Three focus group interviews were held. The first interview was performed by a moderator (ELS) with prior experience of leading focus group interviews. An assistant (VM) took notes during the interviews in order to recall impressions of non-verbal communication between the participants during the analysis. The researchers switched roles in the second and third interviews. All three interviews were performed using a semi-structured interview guide.

### Participants

The GPs in the focus groups were recruited to the study through an invitation letter. In Skåne, GPs from both public and private health care centres have the possibility to meet regularly in previously established continuing medical education (CME) groups to discuss patient cases or different medical, practical or scientific issues [19]. Because of the assumed difficulty in creating new groups, we strategically invited all the pre-established CME groups in Skåne to participate in the study. The groups usually contain 6–12 GPs of different age, gender and experience, from different public and private health care centres. The groups are used to interacting and debating, and feel comfortable expressing and sharing opinions. The invitation letter, sent by e-mail, contained information about the aim of the study and an informed consent form, and offered the possibility to perform the interviews at the CME group’s regular time and place of meeting.

Interview questions were created with an emphasis on the following themes:

- Attitudes towards guidelines
- The impact of using guidelines on the doctor-patient relationship.

### Analysis

The interviews were audio recorded, transcribed verbatim and studied by the first and last authors using thematic content analysis [20,21]. After the transcribed interviews and additional notes had been read, the text was divided into meaning units and condensed. Units with similar content were compiled into different sub-categories, categories and themes, and the results were discussed until a consensus was reached. The method is conventional inductive content analysis with codes and categories derived from data during analysis [22].

### Ethical approval

The study has received ethical approval from the Regional Ethical Review Board in Lund (case no: 2013/392).

## Results

Three focus group discussions were held with a total of 17 participants, with 5, 5 and 7 GPs in groups 1, 2 and 3, respectively. The characteristics of the participants are shown in Table 1.

**Table 1:** **Characteristics of the participants**

**Focus group**	**Participant**	**Sex**	**Age**	**Age, median**	**Years of practice**	**Years of practice, median**	**Practice**
1	A	F	57	54	20	20	Public
	B	F	54		25		Public
	C	F	50		15		Public
	D	F	45		16		Public
	E	F	58		30		*Private*
2	A	M	53	53	10	10	Public
	B	F	61		33		Public
	C	F	64		35		Public
	D	F	34		4		Public
	E	F	38		3		Public
3	A	F	35	40	7	5	Public
	B	F	48		5		Public
	C	M	41		10		Public
	D	F	48		5		*Private*
	E	M	35		5		Public
	F	F	40		8		Public
	G	M	33		2		Public

An example of the text condensation in meaning units is shown in Table 2.

**Table 2:** **Example of text condensation and coding**

**Theme**	**GP-related influencing factors**		
**Category**	Beliefs about adherence to guidelines		
**Final coding**	Reported adherence behaviour in everyday practice		
**Initial coding**	High adherence if guidelines similar to own experience	Lower adherence if more frequent changes to guidelines	High adherence when feeling unsure
**Condensed meaning unit**	In the case of migraine drugs, when I did not have enough experience to say that the more expensive drugs were better, I supported my argument with the guidelines.	It was decided that the insulin kind would change to another, cheaper one, and soon afterwards it would change back again, but I have learned from previous experience and have not changed anything yet.	When I feel unsure I stick to the guidelines.
**Meaning unit**	“… and an area where I’ve benefited from them … (guidelines) … in agreement with the patient or against the patient’s will … is when they want migraine drugs, triptans, more expensive ones … and when I didn’t have enough experience to say that that the more expensive ones were better, I supported my argument with the guidelines then …”	“… we were supposed to change from the usual insulin that we had used many years to a cheaper one, and it is a lot of work if you are going to change it for all patients, and then after a couple of months they lowered the price of the first one, so there was no difference any more. But I have some previous experience and have not changed anything yet, but will wait and see what happens.”	“You feel sometimes that you should be more informed, but if I feel unsure I stick to the guidelines”.

Seven categories emerged during the coding process (Table 3). The categories were grouped into two main themes: **GP-related influencing factors** and **External influencing factors**.

**Table 3:** **Categories and themes**

**Categories**	**Themes**
Expectations and perceptions about existing local guidelines	**GP-related influencing factors**
Knowledge about evidence-based prescribing	
Trust in development of guidelines	
Beliefs about adherence to guidelines	
Patient-related aspects	**External influencing factors**
Drug industry-related aspects	
Health economic aspects	

### GP-related influencing factors

The first category included in this theme was ***“Expectations and perceptions about existing local guidelines”*** (Table 4). GPs stated unanimously during the discussions that they perceive guidelines as a form of support, that they do not feel bound by them but feel safe when using them. They also stated that they feel free to deliberately deviate from guidelines if necessary and expected the existence of second and third choice drug on the list of recommended drugs. Several GPs expressed a belief that the aim of guidelines was cost containment and also that guidelines focus primarily on drug costs and not on the patient. A majority of GPs perceived the local guidelines as time saving.

**Table 4:** **Categories and quotations for the theme “GP-related influencing factors”**

**Category**	**Quotations**
**Expectations and perceptions about existing local guidelines**	“… and then I feel free, that if it doesn’t work with these basic drugs, it’s not a problem to prescribe something else …”
	“… it is easy to check with the list … and maybe I don’t have the same critical judgement as before, but on the other hand I save time, because I perhaps wouldn’t have had the time anyway …”
**Knowledge about evidence-based prescribing**	“It has a lot to do with our stress, that we don’t have the time to sit and read *Läkartidningen* ^a^ or to look at our drugs, what there is and what the options are … it is about our time … that we actually don’t have time to do it. Instead it is easier to reach for something like this … just as you say …”
	“A good thing to bring up, I think, is the new electronic medical records system, PMO, that [the prescriptions] are there, so it is very easy to prescribe a recommended drug, which is very positive”.
	“I didn’t even know that the guidelines were there, where do you find them?”
**Trust in development of guidelines**	“… then I wonder a little bit…they are after all human beings … these groups who sit and write the guideline, I mean … we don’t know how active and good the doctors in these groups are …”
	“…then I wonder, why does it have to be local, does it have to be different … in every region … are the patients different?”
	“The background information? Yes, it is very robust and good. If I didn’t have that book I perhaps wouldn’t have been as … satisfied or had the same confidence, because I can … read about what they considered and how the drugs work”.
	“But it feels quite uncomfortable, because they’re new drugs that we’ve heard so many good things about, and they cost a lot, but you sit there and wonder … well … nobody else tries it …”
**Beliefs about adherence to guidelines**	“A barrier would also be, as I said, a lack of options. It is a barrier to following guidelines, because you don’t know whether it will work in the next step …”
	“Sometimes they come with different pills from the hospital, which they don’t need, and then we are supposed to withdraw them and prescribe the recommended ones. I can say that often the patient goes along with it, because I have the book there with the guidelines …”
	“It is actually aimed at GPs; hospital doctors don’t read it.” “Sometimes it feels that they don’t know what we are doing … they are supposed to follow the guidelines for the drug … but I don’t think they do…”
	“Yes, I agree with you, C … if a patient has a drug that works I don’t change it either just because they change the guidelines. Because … I don’t want to make the poor old patients more confused than they already are…”

^a^A Swedish-language medical periodical.

The second category was **“*Knowledge about evidence-based prescribing”***. Although participants unanimously agreed that drug treatment should be evidence based, all of them reported a lack of time to self-inform about new drugs or therapy recommendation changes. They also revealed different levels of knowledge about the existence of and use of IT-based guidelines. All the GPs reported easy access to guidelines in a paper folder and welcomed the annual DTC-arranged conference with information about guidelines.

The category ***“Trust in development of guidelines”*** showed that all the GPs welcomed the detailed background information following the guidelines. They reported that they felt more prone to adhere to guidelines when informed about the decision process presented by the DTC based on background research about the recommended drugs. A historic change in attitude towards the DTC among GPs was described, with a more positive attitude and greater trust during recent years. Different levels of knowledge about how the guidelines were formed were revealed; however, trust in the DTC was described as being more important. Several GPs expressed curiosity about the structure of the local DTC and its work on developing guidelines.

A recurrent subject, spontaneously discussed by all three groups, was the existence of local guidelines, with emphasis on the risk for unequal care in Sweden. Even if most GPs agreed about the importance of local experience and increased adherence if guidelines were local, some GPs were concerned with different prescribing habits in different regions and the consequences for the patients, such as different access to expensive drugs.

An interesting aspect is that most of the GPs reported caution with trying new drugs, using patient safety as an argument; however, they agreed that the introduction of new therapies might be delayed if primary care waits for secondary-care specialists to prescribe them, e.g. drugs for treatment of diabetes.

The category ***“Beliefs about adherence to guidelines”*** revealed several dimensions with attitudes towards both GPs’ own and others’ prescribing. Most of the GPs agreed that prescriptions should be based on guidelines. The frequency of guideline updates was discussed and some GPs requested more frequent updates than the current annual ones, with faster introduction of new drugs. However, a majority of GPs reported lower adherence if recommendations changed often.

The first focus group had longer experience in primary care practice (Table 1). The second group included physicians with a great range of experience and the debate within the group was dominated by the more experienced GPs, the youngest having a more passive and confirmatory role. The third group, which included younger physicians with shorter experience, expressed a greater concordance of opinions regarding the acceptance of guidelines as a prescribing tool, explaining it as the result of early training in following evidence-based practice.

A majority of the participants expressed concerns about having difficulties managing other doctors’ prescribing and feeling uncomfortable changing prescriptions according to guidelines if the patient had multiple prescribers. Some GPs described strong beliefs that guidelines were directed to primary care and were not compulsory for hospital doctors or private secondary care specialists.

### External influencing factors

The first category in this theme was ***“Patient-related aspects”*** (Table 5), where patient safety was described as an important factor influencing the prescribing decision. A majority of GPs reported deviation from guidelines if a drug caused adverse drug reactions or if changing the drug would result in lower compliance with treatment. Patient safety was ranked as more important than maintaining a good patient-doctor relationship, e.g. regarding prescription of antibiotics. GPs reported the belief that patients’ expectations might sometimes be different from those of doctors; however, it was unusual for patients to be unwilling to change drug therapy. A majority of GPs reported a belief that patients have more trust in drugs prescribed in hospitals, leading to difficulties in changing therapy according to guidelines in primary care. Some GPs felt uncomfortable about not being able to always meet patients’ expectations. GPs also believed that patients might feel safe knowing that GPs adhere to guidelines but that patients usually have little knowledge about the existence of guidelines. Patient-adapted information about guidelines was believed to increase compliance and safety, to benefit the patient-doctor relationship, and to be a better alternative to drug advertising from the pharmaceutical industry. The importance of dialogue with the patient was a recurrent issue and a majority of the GPs reported that guidelines facilitated the patient-doctor relationship.

**Table 5:** **Categories and quotations for the theme “External influencing factors”**

**Category**	**Quotations**
**Patient-related aspects**	“Yes, you should never experiment with patients … or expose them to risk of injury. It is very important. This is why I think that we GPs are very careful with new drugs. I prefer to wait a while with a new drug before I prescribe it …”
	“You might think so, but the patient may think differently …”
	“ … I think it is very important not to give in, at least in those cases with tetracycline versus penicillin, it feels important to explain to the patient the risk of bacterial resistance and so on … so there you can compromise a bit on the patient-doctor relationship …”
**Drug industry-related aspects**	“A conflict arises sometimes. Some patients are so well-read and influenced by the media and sometimes want another drug and … insist …”
	“We don’t know anything about that. We don’t know if somebody there is on Pfizer’s board … or is biased …”
	“… and then you think about how life was before the [local] guidelines even existed. … we were drug industry indoc … formed … (laughs)”
**Health economic aspects**	“…I think that it is OK to save money on things you can save money on … maybe to be able to do more tests of that kind or something else … the budget is not unlimited, so I usually think that this is not a problem.”
	“… but there is a lot of focus on economy here, more focus on economy than on the pharmacological benefit compared to other drugs … so from that point of view it is highly controlled …”
	“I am not really sure if the economy part motivates us …”
	“The goal is to save money, I suppose, and more and more of the drug costs are transferred to the primary health care centres … so of course it matters …”

The category ***“Drug industry-related aspects”*** included GPs’ statements about difficulties in managing direct-to-consumer commercials about drugs and their impact on patients. Some GPs wondered about possible influences of the drug industry on the local DTC. The GPs described an historical change in how GPs get information about new drugs as a shift from information from the drug industry to objective academic detailing from the DTC.

The category ***“Health economic aspects”*** included GPs’ statements about how economic considerations should or should not influence adherence to guidelines. The GPs expressed a feeling of economic responsibility for both patients and society, revealing a two-sided attitude and a dilemma faced in the prescribing situation.

Some GPs reported a belief that guidelines take cost efficiency into account more than patients’ individual needs. A subject largely discussed in the groups was economic aspects in forming the guidelines. GPs expressed both reluctance and understanding, describing the economic perspective as both a barrier and a motivator for adherence to guidelines. A majority of the GPs reported understanding of the necessity of priorities in primary care, but also a negative attitude towards the influence of economic terms on the prescribing decision. The core motivators for adherence to guidelines were found to be the time-saving aspect, trust in evidence-based market-neutral guidelines, patient safety and the feeling of economic responsibility for both patients and society. Main barriers to adherence were cost containment as a decision factor in developing guidelines, multiple prescribers with unclear responsibility for patients’ medication lists and drug industry information addressed directly to the public.

## Discussion

### Main findings

We found two main themes describing GPs’ attitudes towards local treatment guidelines: **GP-related influencing factors** and **External influencing factors**.

The attitudes were grouped into seven main categories: ***Expectations and perceptions about existing local guidelines*,*Knowledge about evidence-based prescribing*,*Trust in development of guidelines*,*Beliefs about adherence to guidelines*,*Patient-related aspects*,*Drug industry-related aspects*,*and Health economic aspects***. To rely on evidence-based guidelines and the time-saving benefit of using local guidelines were described as key motivating factors for adherence, suggesting that understanding of the development process and easy access to local guidelines are factors with big implications for future guideline design and implementation. Patient safety was reported to be more important than adherence to guidelines or maintaining a good patient-doctor relationship. GPs described both positive and negative attitudes to cost containment, which was perceived both as a motivating factor and a barrier for adherence to guidelines. GPs expressed concerns about difficulties with adherence to guidelines while managing drugs from other prescribers and drug industry information addressed directly to the public.

#### Strengths and limitations

Previous research has focused on GPs’ adherence to nationally developed guidelines [9,23,24], using a questionnaire-based approach. We found no previous qualitative research with focus groups studying GPs’ attitudes towards adherence to local guidelines, which is a novel aspect of this study.

Focus groups as a qualitative research method have been approached from different theoretical point of views. For instance, social contextual constructivist researchers address the “process” of interaction among individuals, in a specific context in which people live and work, and recognize that the researcher’s own background shapes their interpretation [25]. Social contextual constructivists emphasize the importance of the researcher’s reflexivity and the context-dependent method. The realist theoretical framework focuses on reliability and validity in qualitative studies in order to present the presumed only existing reality [26]. Methodological tensions have been described between contextual constructionist and realist theory frameworks behind focus groups [27]. According to contextual constructionism, pre-existing groups may provide “naturalistic” exchanges by encouraging participation by people who are reluctant to be interviewed or feel they have nothing to say [18]. From a realistic point of view pre-existing groups should be avoided given their potential for bias [28]. We believe that the strategic use of pre-existing groups of GPs with different experience and gender, working at both private and public health care centres and with previous contact and familiarity with the debate within the group was a strength of the study. Five to seven participants are recommended for focus groups and we managed to include at least five GPs in each group. Since the aim of the study was to understand the factors that affect adherence to guidelines, and not to generalize the results, we consider 17 participants to be satisfactory.

There was a general concordance of opinions within the groups; however, the interviews created a debate allowing the participants to express a great variety of attitudes towards particular issues, such as the frequency of updates and economic aspects, which increased the credibility of the results. These interesting aspects of different group dynamics suggest that even if heterogeneous groups might facilitate a debate, great variation in professional experience is a possible limiting factor, less experienced doctors being more hesitant in expressing their opinions. However, including GPs with different levels of experience might have increased the transferability of the results of this study.

One of the researchers (VM) knew 12 of the 17 participants as colleagues, which could be both an advantage and an obstacle. Her role as a GP might have encouraged free debate due to an assumed mutual understanding of the cultural context the participants worked in. However, no specific reactions on this matter were discussed or observed. VM is also a member of the local DTC and her role as an objective researcher in the study with no links of an economic or employment nature was stressed prior to the interviews. She also explained her role as a researcher in order to avoid addressing debate questions related to her pre-understanding of the discussed topic. However, even if data collection and analysis were performed with objective reflexivity and with continuous awareness of her pre-understanding of the topic taken into account, this might have been a limitation of the study. The second researcher present during the interviews (ELS) had a background as a social worker and had no previous contact with the participants or pre-understanding of the studied subject. Due to the researchers’ different levels of pre-understanding, they switched role during the interviews. This might have served as a strength by increasing the dependability of the results.

The GPs in this study reported strong adherence. However, international data show that GPs overestimate their adherence to guidelines, suggesting that self-reported adherence might not correlate well with actual prescribing behaviour and should not be used as the sole measure of guideline adherence [29]. No prescribing data were collected as we did not aim to assess prescribing behaviour. This means that we cannot draw any conclusions from this study about Swedish GPs’ adherence to local guidelines.

#### Comparison with existing literature

As previously described in other studies, Swedish GPs often believe that treatment guidelines are useful in practice and generally have a positive attitude to them [24]. They see prompt and pragmatic benefits as a strong motivating factor, though differences exist between GPs [15]. However, a meta-analysis of qualitative research shows that GPs’ attitudes towards treatment guidelines may be influenced by the purpose of the guidelines and that trust might be more important than access when implementing them [23], similar to the results in our study.

The GPs in this study did not report that adherence to guidelines would lead to a poorer patient-doctor relationship. The results are different from international data. A Canadian study showed that the use of recommendation lists based on a controlled replacement model led to poorer patient contacts, increased stress for doctors and increased the frequency of contacts with the healthcare system [30]. A British study showed that a strong feeling of clinical autonomy and resistance to economic decisions caused a sceptical attitude towards clinical guidelines and that emphasis on cost-effectiveness might be counterproductive [31]. The participants in our study reported concerns about the negative effect of economic aspects in forming guidelines, findings similar to those of other studies [32]. However, cost containment was not frequently reported to be a negative factor in decision making or to affect the patient-doctor relationship. These findings, unlike those from other studies, might be due to the unique social and professional context Swedish GPs work in, in larger multi-professional surgeries with shared economic responsibility. However, the impact of different organisational contexts on GPs’ attitudes towards adherence to guidelines was not studied in this paper. The results might also mirror the historical change in attitudes towards drug information. The participants described a paradigm shift in GPs’ attitudes towards drug information sources during recent decades, with an increasingly positive attitude towards academic detailing provided by the local DTC instead of drug industry-supplied information. Younger GPs reported higher adherence to local guidelines. This is consistent with findings from a recent Swedish study [33], which showed that Swedish GPs who were older or had more experience were more positive to drug industry-supplied information than younger GPs. Frequent changes in recommendations were viewed both positively and negatively, with great variation between the participants. GPs reported trust in evidence-based guidelines, but also interest in the operations of the local DTC. However, they did not express opposition to a top-down managerial initiative about prescribing quality. Our findings indicate that transparency in forming guidelines, such as information about the structure and methods of the local DTC together with regular academic detailing about the guidelines, might increase confidence in the local DTC and thus enhance adherence. A recent Canadian study showed that GPs believe that involvement of frontline practitioners in developing guidelines might facilitate implementation by maximizing the objectivity of recommendations [34]. This suggests that increased knowledge among Swedish GPs about the structure of DTCs, which involve GPs in the development of guidelines, might further enhance adherence.

GPs described the patient-doctor encounter, with emphasis on informing the patient about guidelines if necessary, as very important. This factor has been found to enhance adherence to guidelines, such as recommendations for prudent antibiotic prescribing [35].

## Conclusions

The Swedish GPs in this study reported that patient safety, the time-saving aspect, trust in evidence-based market-neutral guidelines and the patient-doctor encounter, with emphasis on informing the patient were core motivators for adherence to guidelines. Main barriers to adherence were cost containment as a decision factor in developing guidelines, multiple prescribers with unclear responsibility for patients’ medication lists and drug industry information addressed directly to the public. Future studies should explore the need for transparency in forming and implementing guidelines, which might potentially increase adherence to evidence-based treatment guidelines in primary care.

## Abbreviations

GP: General practitioner
DTC: Drug and therapeutic committee
CME: Continuing medical education
